# X-linked severe combined immunodeficiency with Down syndrome identified by newborn screening

**DOI:** 10.70962/jhi.20250057

**Published:** 2025-07-14

**Authors:** Yusuke Imanaka, Takaki Asano, Kosuke Noma, Fumiaki Sakura, Norifumi Nishioka, Yoko Mizoguchi, Takahiro Kamiya, Hirokazu Kanegane, Satoshi Okada

**Affiliations:** 1 Department of Pediatrics, Hiroshima University Graduate School of Biomedical and Health Science, Hiroshima, Japan; 2 Department of Genetics and Cell Biology, Research Institute for Radiation Biology and Medicine, Hiroshima University, Hiroshima, Japan; 3 Department of Pediatrics, National Hospital Organization Kure Medical Center and Chugoku Cancer Center, Hiroshima, Japan; 4 Department of Pediatrics and Developmental Biology, Institute of Science Tokyo, Tokyo, Japan; 5 Department of Child Health and Development, Institute of Science Tokyo, Tokyo, Japan

## Abstract

Suspected false positivity in newborn screening delayed diagnosis of X-linked severe combined immunodeficiency in an infant with trisomy 21, underscoring the diagnostic challenges of coexisting genetic conditions and the need for timely immunological evaluation and intervention to prevent life-threatening infections.

## Teaser

This is the first reported case of Down syndrome complicated by severe combined immunodeficiency successfully treated with hematopoietic stem cell transplantation. While Down syndrome can lead to low TREC levels in newborn screening, which typically recover spontaneously, careful follow-up is indispensable for potential risk.

Severe combined immunodeficiencies (SCIDs) are rare, life-threatening inborn errors of immunity (IEI) with impaired T cell differentiation and/or function, often with B/natural killer cell abnormalities. Most SCID patients are asymptomatic at birth but develop recurrent infections within the first few months. Without curative treatment, including allogeneic hematopoietic cell transplantation (HCT), most do not survive beyond the first year.

Recently, quantification of T cell receptor excision circles (TRECs), a DNA byproduct of T cell recombination, has been widely used to detect impaired T cell development and facilitate SCID screening. However, conditions such as prematurity and low birth weight are known to result in low TREC values; therefore, careful interpretation in these newborns is required. In Japan, newborn screening (NBS) using TRECs was introduced in 2017, enabling earlier SCID detection.

Down syndrome (DS) is a genetic disorder caused by the presence of an extra copy of chromosome 21, characterized by distinctive facial features, developmental delays, and varying degrees of intellectual disability. Individuals with DS sometimes exhibit increased susceptibility to certain pathogens due to immune dysfunction. Additionally, low TRECs are occasionally observed in individuals with DS during NBS, though typically recover spontaneously. This can complicate diagnosis, particularly when a patient with DS has SCID or another IEI. To date, only one case of DS complicated by suspected SCID without a genetic diagnosis has been reported (1). Moreover, there have been no reports of HCT for such cases. This is the first reported case of DS complicated by SCID that was successfully treated with HCT.

**Figure 1.**
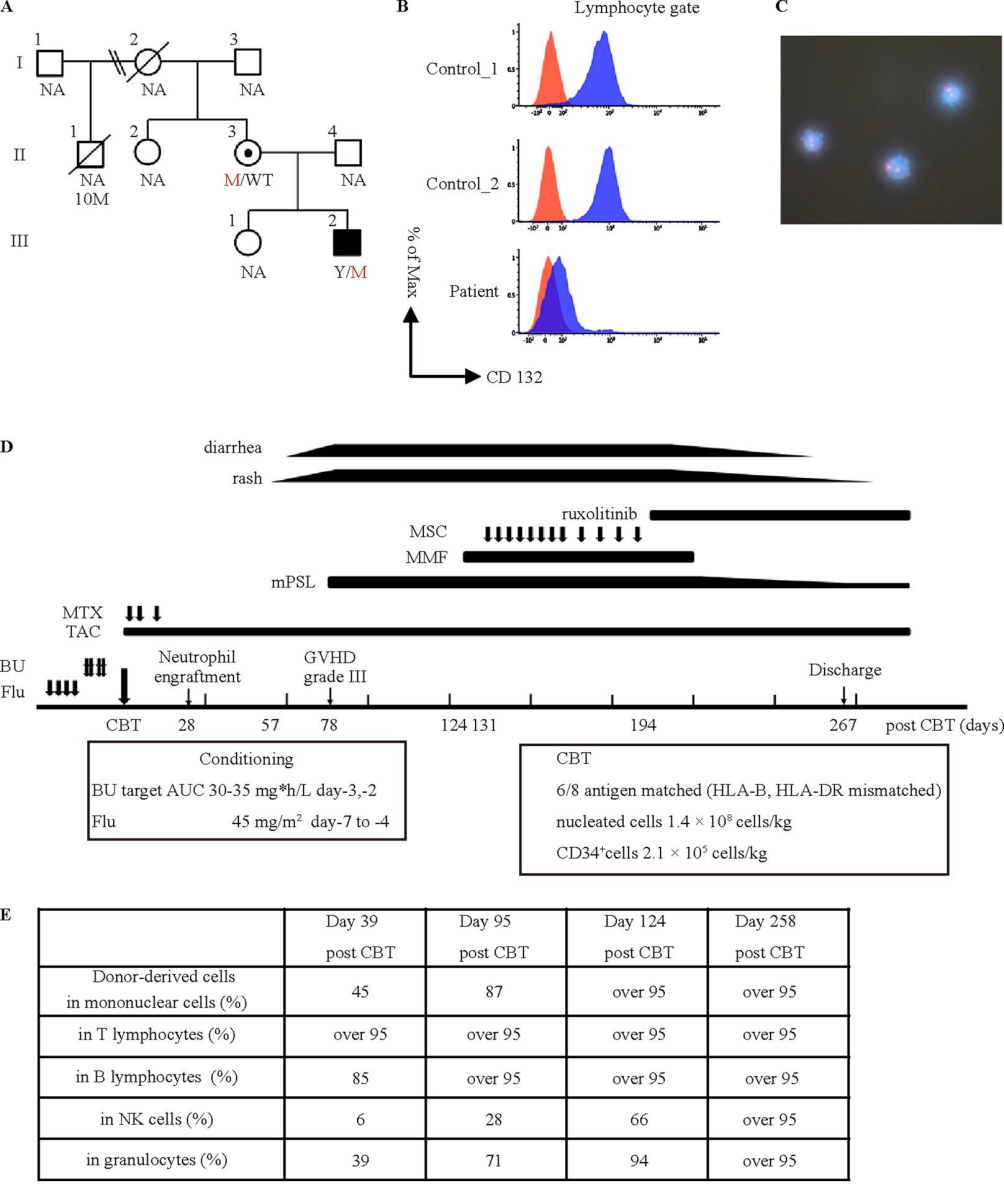
**Genetic, immunological, and clinical features in a patient with X-linked SCID. (A)** Familial segregation. The arrow indicates the proband. M, *IL2RG* variant, c.455-2A>G on the X chromosome; WT, wild type of *IL2RG*; Y, Y chromosome; NA, not available. **(B)** Flow cytometric analysis of CD132 expression on lymphocytes from patient and healthy control PBMCs. Cells were surface stained with anti-CD132-APC (clone TUGh4; BioLegend) for 20 min at room temperature. Lymphocytes were pre-gated by FSC/SSC. Data were acquired using a BD FACSVerse flow cytometer. Red represents isotype control, and blue represents CD132 expression. **(C)** XY-fluorescence in situ hybridization (FISH) of T cells. T cells were isolated from the patient’s peripheral blood by cell sorting using a FACS Aria flow cytometer. XY-FISH shows engrafted maternal T cells in the peripheral blood. His original T cells have green and red signals (XY chromosomes, not expressed in this figure), and engrafted maternal T cells have two red signals (XX chromosomes). **(D)** Clinical course of the patient after umbilical CBT. BU, busulfan; Flu, fludarabine; MTX, methotrexate; TAC, tacrolimus, mPSL, methylprednisolone; MMF, mycophenolate mofetil; MSC, mesenchymal stem cells. **(E)** Donor chimerism analysis in each cell fraction. This is the first reported case of DS complicated by SCID successfully treated with hematopoietic stem cell transplantation. While DS can lead to low TREC levels in NBS, which typically recover spontaneously, careful follow-up is indispensable for potential risk. PBMC, peripheral blood mononuclear cells, FSC, forward scatter, SSC, side scatter, AUC, area under the curve.

The patient was born to non-consanguineous Japanese parents at 35 wk of gestation, with a birth weight of 1,723 g. He was diagnosed with DS based on phenotype with trisomy 21. Multiple comorbidities were identified after birth, including atrial septal defect, ventricular septal defect, and transient abnormal myelopoiesis. There was no history of severe disease in his parents or 8-year-old sister. However, it was later discovered that the mother’s maternal half-brother (II.1) had died before 8 mo of age due to recurrent severe infections (Fig. 1 A). NBS for SCID at birth revealed a low TRECs level of 4 copies/μl. Genetic testing was not performed at that time, as it is known that in DS, naïve T cells are reduced due to abnormal thymic development and function, leading to low TREC levels. It was expected that TREC levels would increase over time. He was managed in the neonatal intensive care unit with infection control measures, including discontinuation of breastfeeding; avoidance of live vaccines; and administration of sulfamethoxazole/trimethoprim, itraconazole, and immunoglobulin.

He was discharged at 3 mo without infections. At home, he remained infection-free, continuing the preventive therapy. Contrary to expectations, his TREC levels remained low, and lymphocyte counts were consistently low, ranging from 800 to 1,000/μl, with naïve helper T cells comprising only 0.2–0.9% of total lymphocytes. Targeted panel exome sequencing performed at 11 mo identified a novel hemizygous *IL2RG* variant, c.455-2A>G, which has inherited from asymptomatic mother (Fig. 1 A). Additionally, flow cytometry revealed a deficiency of CD132 (common γ chains) in peripheral blood (Fig. 1 B). Fluorescence in situ hybridization assays detected the maternal T cells in the patient’s peripheral blood (Fig. 1 C), leading to a diagnosis of X-SCID, despite not strictly meeting diagnostic criteria. Consequently, he was hospitalized in preparation for HCT.

At 14 mo, he underwent umbilical cord blood transplantation (CBT) from a human leukocyte antigen (HLA) two-locus mismatched (HLA-B and HLA-DR) donor using a reduced-intensity conditioning regimen, which included busulfan and fludarabine (Fig. 1 D). The number of infused nucleated cells was 1.4 × 10^8^ cells/kg, and CD34^+^ cells were 2.1 × 10^5^ cells/kg. Prophylaxis for graft-versus-host disease (GVHD) consisted of tacrolimus and short-term methotrexate. Neutrophil engraftment was achieved on day 28 after CBT, with CD3-positive T cells detected in peripheral blood by flow cytometry. Flow cytometry confirmed CD132 expression. Although donor chimerism on day 39 revealed mixed chimerism, the proportion of donor-derived cells gradually increased over time (Fig. 1 E). Ultimately, complete donor chimerism was achieved in all cell fractions by day 250.

On day 57, a pruritic rash appeared, spreading by day 73. Diarrhea appeared concurrently and worsened on day 78. A gastrointestinal biopsy revealed findings consistent with GVHD, leading to the diagnosis of grade Ⅲ GVHD (stage 2 for the skin and gut). Both skin and gastrointestinal symptoms improved with intravenous methylprednisolone; however, symptoms worsened when the dose was reduced. Mycophenolate mofetil (400 mg/day) was initiated on day 124, followed by mesenchymal stem cells therapy starting on day 137 for steroid-dependent GVHD. Despite these interventions, GVHD symptoms continued to worsen with methylprednisolone tapering. Following the introduction of ruxolitinib on day 194, GVHD symptoms did not recur despite continued steroid reduction. Notably, after introducing ruxolitinib, developmental delay improved compared with before treatment. This improvement, particularly in motor development, was noted by the mother, who was closely monitoring him. Aside from GVHD, no severe transplant-related complications occurred, and he was discharged on day 267.

HCT is the curative treatment for SCID, with outcomes largely dependent on the timing of transplantation and the presence of active infections. The 5-year survival rate is 94% for patients who have undergone HCT before 3.5 mo of age without active infection, whereas it decreases to 50% for those with active infections or who receive HCT after 3.5 mo. Therefore, it is well established that both early detection and timely HCT are critical for improving SCID outcomes (2). In our case, SCID was diagnosed at 11 mo due to the diagnostic challenges, leading to a delayed HCT at 14 mo, later than typically observed in standard SCID cases. Although the HCT was successful partly due to the absence of severe or active infection prior to HCT and the ability to proceed without active infection, earlier consideration and diagnosis might have allowed for an earlier HCT.

TRECs are circular DNA fragments produced during T cell receptor gene rearrangement in developing T cells and serve as a marker for newly generated T cells. Since TREC levels are low in conditions where T cell production is impaired, such as SCID, screening using TRECs can aid in early detection of SCID. However, TREC levels can also be low in prematurity or congenital heart disease, and reduced levels do not necessarily indicate immunodeficiency in these cases. In DS, structural and functional abnormalities of the thymus, such as defects in thymic epithelial cells and thymic atrophy, are frequently observed. These abnormalities impair the differentiation and maturation of T cells within the thymus, leading to a reduced number of newly generated naïve T cells, including naïve helper T cells and, as a result, lower TREC levels. In a screening of 80,791 newborns in Aichi prefecture, Japan, 112 cases with low TREC levels (cutoff: 31 copies/μl) were identified, 12 of whom had DS (3). None of these 12 DS cases with low TREC levels were diagnosed with IEI, including SCID. However, as in our case, there is a possibility that a patient with DS and low TRECs values may have an IEI, such as SCID, highlighting the need for careful follow-up. Although DS is associated with reduced naïve helper T cells, the markedly low count in this case likely reflects the additional impact of SCID. SCID in DS is extremely rare; statistical comparison with DS alone is currently not feasible.

Our patient developed steroid-dependent GVHD. Although ruxolitinib facilitated steroid dose reduction, overall GVHD management remained challenging. The refractory GVHD was attributed to transplantation from an HLA two-locus mismatched donor. Additionally, the patient’s underlying DS may have contributed to complications. A report on 11 DS patients with acute lymphoblastic leukemia who underwent HCT revealed that 10 patients experienced severe infections, and 6 had major respiratory disorders. Acute GVHD of grade II–IV and grade III–IV was observed in 9 cases (81.8%) and 7 cases (63.6%), respectively (4). DS is associated with high incidence of treatment-related complications and possibly a risk factor for severe GVHD. Therefore, careful consideration of risks is essential when performing HCT in DS patients.

Recent studies have reported overexpression of interferon receptors in DS patients associated with chronic interferon hyperactivity and systemic inflammation. This hyperactivity has been implicated in a pro-inflammatory phenotype and dysregulation of major growth signaling and morphogenic pathways. Moreover, JAK inhibitors have shown potential efficacy in alleviating various DS symptoms, including growth and developmental delay, by suppressing interferon hyperactivity as reported in mouse models (5). In our case, interestingly, an improvement in motor development was observed following the initiation of ruxolitinib. While this improvement may be attributed to the resolution of GVHD-related symptoms, the potential direct effects of ruxolitinib cannot be excluded. While this is a single case report without pre-treatment-blinded assessment, the observed motor improvement suggests a potential impact of JAK inhibition. Future studies should use standardized motor scales and blinded evaluations. This is the first reported case of DS complicated by SCID successfully treated with HCT. While DS can lead to low TREC levels in NBS, which typically recover spontaneously, careful follow-up is essential due to the potential risk of underlying IEI such as SCID.

## Data Availability

The data are available from the corresponding author upon reasonable request.
